# Steered Molecular Dynamics Simulations Reveal the Likelier Dissociation Pathway of Imatinib from Its Targeting Kinases c-Kit and Abl

**DOI:** 10.1371/journal.pone.0008470

**Published:** 2009-12-24

**Authors:** Li-Jun Yang, Jun Zou, Huan-Zhang Xie, Lin-Li Li, Yu-Quan Wei, Sheng-Yong Yang

**Affiliations:** State Key Laboratory of Biotherapy and Cancer Center, West China Medical School, Sichuan University, West China Hospital, Chengdu, People's Republic of China; Griffith University, Australia

## Abstract

Development of small molecular kinase inhibitors has recently been the central focus in drug discovery. And type II kinase inhibitors that target inactive conformation of kinases have attracted particular attention since their potency and selectivity are thought to be easier to achieve compared with their counterpart type I inhibitors that target active conformation of kinases. Although mechanisms underlying the interactions between type II inhibitors and their targeting kinases have been widely studied, there are still some challenging problems, for example, how type II inhibitors associate with or dissociate from their targeting kinases. In this investigation, steered molecular dynamics simulations have been carried out to explore the possible dissociation pathways of typical type II inhibitor imatinib from its targeting protein kinases c-Kit and Abl. The simulation results indicate that the most favorable pathway for imatinib dissociation corresponds to the ATP-channel rather than the relatively wider allosteric-pocket-channel, which is mainly due to the different van der Waals interaction that the ligand suffers during dissociation. Nevertheless, the direct reason comes from the fact that the residues composing the ATP-channel are more flexible than that forming the allosteric-pocket-channel. The present investigation suggests that a bulky hydrophobic head is unfavorable, but a large polar tail is allowed for a potent type II inhibitor. The information obtained here can be used to direct the discovery of type II kinase inhibitors.

## Introduction

Protein kinases are enzymes essential for cell signal transduction, which regulate a variety of physiological processes including metabolic, cell cycle, apoptosis and cell differentiation [1]–[3]. Dysregulation of protein kinases might lead to some pathological changes, for example, cancer, diabetes, and various autoimmune diseases [4], [5]. Thus protein kinases have been thought as central targets for drug discovery. In the past decade, extensive efforts have been made to develop protein kinase inhibitors as potential drugs against a wide range of diseases [6]–[13]. And it is believed that understanding of issues related to the protein kinase structures, mechanisms underlying enzyme activation and the kinase-inhibitor interaction could benefit the discovery of novel kinase inhibitors.

**Figure 1:**
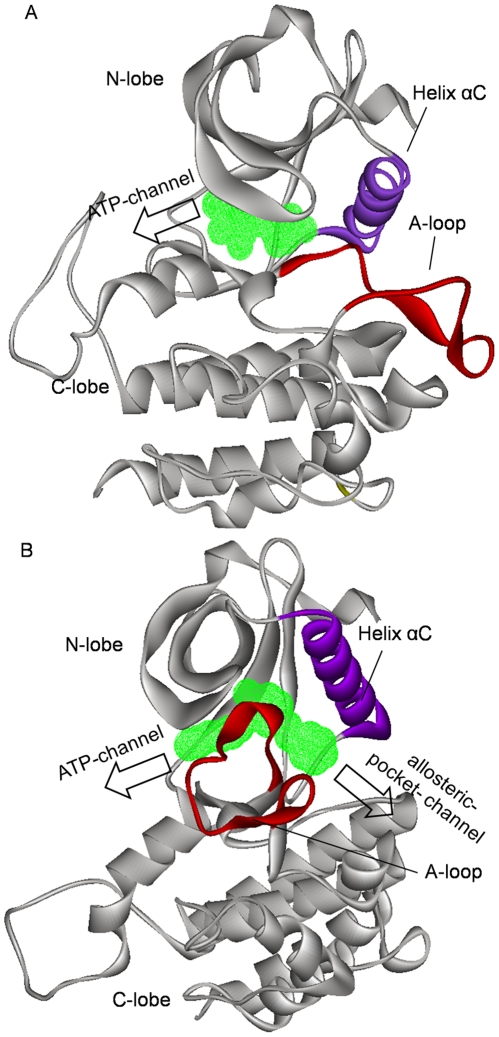
Typical three-dimensional structures of protein kinases shown in Cα ribbon fashion. (A) is for active conformation, and (B) for inactive conformation. Key structural components of the protein are color coded: A-loop in red, helix αC in purple, others in gray. Type I (for the active conformation) and type II (for the inactive conformation) kinase inhibitors are schematically shown in green wire mesh.

All protein kinases share a common catalytic domain, which contains two subdomains: the N-terminal lobe and the C-terminal lobe [14]. The two lobes are connected through a flexible chain (hinge region). The natural substrate ATP is bound in the cleft between the two lobes (the ATP binding pocket). The active loop (A-loop), which belongs to the C-terminal lobe but locates outside of the ATP-binding pocket, directly regulates the enzyme activation through its conformational changes. Majority of small molecule kinase inhibitors reversibly occupy the ATP binding pocket, which means that they are ATP-competitive inhibitors. The ATP-competitive inhibitors can be further classified into two categories, type I and type II [15], [16]. Type I inhibitors target the active form of the kinases, in which the A-loop adopts an extended conformation. Such conformational arrangement of A-loop completely exposes the ATP-binding pocket, hence facilitating the entry/exit of ATP or type I inhibitors (this entry/exit pathway will be called as traditional ATP-channel hereafter, see Figure 1A). Type II inhibitors target the inactive form of kinases and bind to an extended ATP-binding site, in contrast to type I inhibitors. In the inactive form, the A-loop crimples outside of the ATP-binding pocket. This conformation of A-loop shrinks the original entry/exit gate, which hinders the access of ATP and protein substrates to the kinase catalytic site. Another concomitant conformational change is the flip of DFG-motif that locates in the beginning of A-loop, which opens a new hydrophobic pocket (usually called allosteric pocket) in the back of the protein [17] (see Figure 1B). Type II inhibitors often occupy both the original ATP-binding pocket and the allosteric pocket. It appears that there are two possible pathways for the entry/exit of type II inhibitors: one is the traditional ATP-channel and the other one is the allosteric-pocket-channel. Now, a question arises that which one is preferred. X-ray crystal structures of kinase-inhibitor complexes show that the allosteric-pocket-channel might be favored since this channel is relatively wider than the ATP-channel [18], [19]. This hypothesis, however, is inconsistent with the fact that many receptor tyrosine kinases have a juxtamembrane region (JMR), which resides close to the gate of allosteric-pocket-channel in the inactive form of kinases. Even so, our previous study on the JMR dynamics did not deny the allosteric-pocket-channel of type II inhibitors [20]. In order to clarify this mechanism, we need to use molecular dynamics simulations, which is mainly due to the fact that the dissociation of ligands from targeting proteins is governed by the dynamic behavior of ligand-protein complexes that is difficult to handle experimentally.

**Figure 2:**
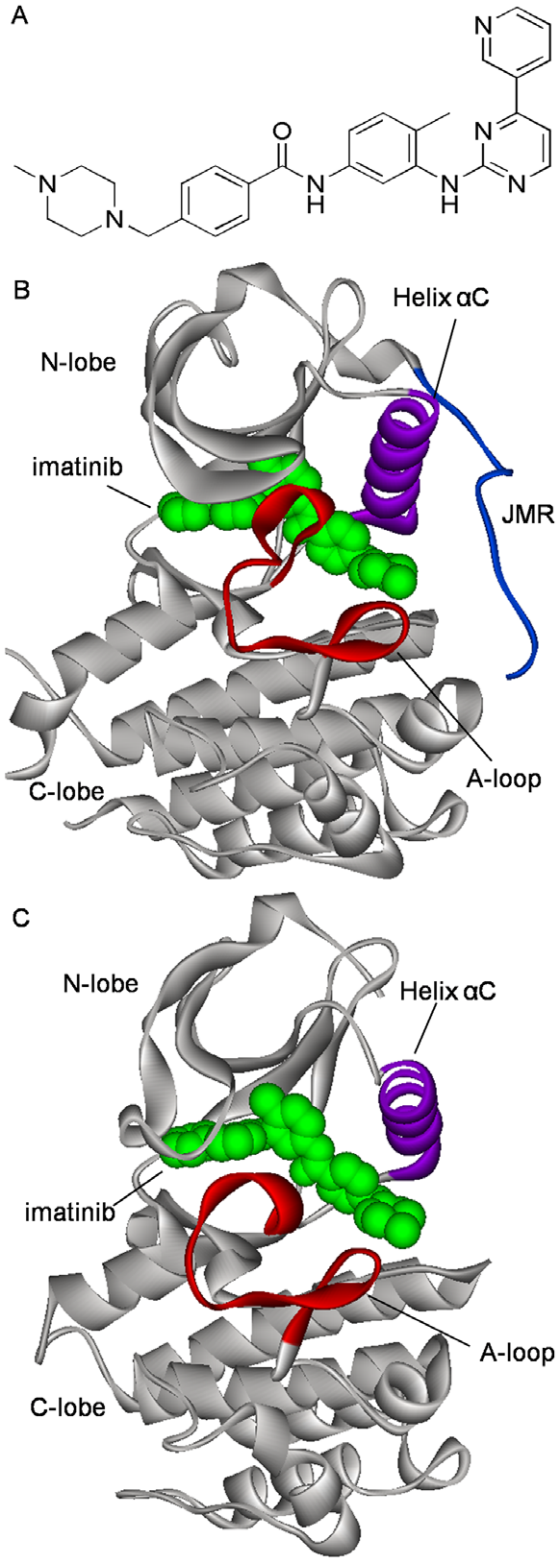
Chemical structure of imatinib and its binding modes to c-Kit and Abl. (A) Chemical structure of imatinib. (B) 3D structure of imatinib-c-Kit complex (PDB entry: 1T46). (C) 3D structure of imatinib-Abl complex (PDB entry: 2HYY). In (B) and (C), protein structures are in Cα ribbon fashion and imatinib in green CPK fashion. Key structural components of the protein are color coded: A-loop in red, helix αC in purple, JMR in blue, others in gray.

In this account, steered molecular dynamics (SMD) simulations [21]–[23] will be employed to explore the possible dissociation channel for type II kinase inhibitors from inactive form of kinases. Despite several methods of molecular dynamics simulations that are currently available for handling this type of rare events (see the section of Models and Methods), SMD has been seen as the most suitable method to address the binding/unbinding issues of biomolecules [24]–[30]. (A brief description about the SMD algorithm, as well as its advantages and disadvantages compared with other approaches, will be given in the section of Models and Methods). Here, we chose imatinib (see Figure 2A) as our model of type II kinase inhibitors; imatinib is the first type II protein kinase inhibitor on the market and has become a reference compound for the kinase community. The cellular targets of imatinib include the Abl/Bcr-Abl protein kinase that is involved in the development of chronic myeloid leukaemia (CML) [31], the c-Kit kinase that is implicated in gastrointestinal stromal tumours (GIST) [32], [33]. Thanks to the advance of structural biology, the crystal structures of c-Kit and Abl in complex with imatinib have been available [18], [19] (see Figure 2B and 2C). Another important reason why we chose both c-Kit and Abl protein kinases as our models is that c-Kit and Abl represent two distinct regulation modes of catalytic activation. Abl is a typical cytoplastic kinase, which activation depends directly on the phosphoralation of TYR412 on A-loop [34]. Contrastively, c-Kit is a typical receptor tyrosine kinase, which activation depends on not only the phosphoralation of the A-loop but also the JMR (TYR1-ASP33) [20], [35]. Since JMR locates around the gate of allosteric-pocket-channel, it seems that the JMR could have some impact on the entry/exit of ligand. In a word, we expected that SMD simulations could provide us with more insights into the entry/exit mechanism of type II inhibitors from the inactive form of protein kinases. We also believe that a detailed understanding of the entry/exit dynamics will ultimately help with the design of new type II kinase inhibitors.

## Methods

### Computational Models

The initial structures for imatinib-c-Kit and imatinib-Abl complexes were prepared based on their corresponding X-ray crystal structures (PDB entry is 1T46 for imatinib-c-Kit and 2HYY for imatinib-Abl). The unresolved residues in imatinib-c-Kit, including TYR547-ASN564, ILE690-THR694, SER753-GLU761, HIS934-ILE935, were constructed by using the homology modeling module Modeler within InsightII (Accelrys, San Diego, CA), in which the reference protein structure was taken from the PDB code 1T45.

### Steered Molecular Dynamics Simulations

SMD is an extended MD simulation method that mimics the basic idea of atomic force microscopy (AFM). SMD allows exploring the binding and unbinding properties of a variety of biomolecules as well as their responses to external mechanical manipulations at the atomic level [36], [37]. It is particularly suitable for the investigation of dissociation of a ligand from its binding protein. In such case, a ligand is forcedly pulled out of the binding site of a given protein along the route that is specified in advance. The pulling force F is applied according to the following equation:
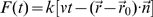
(1)where 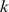 is the force constant, 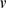 is the pulling velocity, 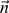 is the pulling direction normal, 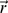 and 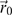 are the position of ligand at time t and initial time. Briefly, if ligand moves forward along the leaving pathway, the 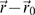 increases and the force decreases; if the pulling force can not displace the ligand, the force will increase because of the increase of t. So, it is possible to draw a profile to express the force required to dissociate the ligand as the function of simulation time, and hence determine the difficulty to dissociate ligand along the different pathway.

Actually several methods that utilize the principles of molecular dynamics to investigate this type of rare processes have been developed. In these methods, some external perturbations are applied to the system in order to accelerate the dynamic process and guide the system toward a target state. Such methods typically include, for examples, targeted molecular dynamics (TMD) [38], [39] and biased molecular dynamics (BMD) [40], [41], in addition to SMD used here. The TMD methodology imposes a time-dependent holonomic or harmonic constraint on the RMSD (root mean square deviation) in order to drive the system to a target structure. BMD provides the least perturbation compared with SMD and TMD, in which the system feels no external force if it moves toward the target and the biasing potential is nonzero only if the system moves away from the target.

In contrast to TMD and BMD, SMD closely resembles the AFM experiment. In addition, the exact target structure is not necessary to know in advance for SMD simulations. These make the SMD very suitable to be used for exploring the dynamics behaviors of binding/unbinding processes of biomolecules. Although SMD provides an approach to investigate the binding/unbinding events, yet the pulling direction of the force in SMD is chosen randomly or by guessing on the basis of structural information, which makes that the force applied to the ligand in such chosen directions may not move it along a favorable pathway. In order to overcome this shortcoming, a generally used strategy is to perform a series of SMD simulations with different directions of pulling force. Dynamic properties of the system can be obtained by averaging those of all the individual simulations. This strategy has also been used in this investigation, which will be mentioned again later.

### Computational Details

All the simulations, including conventional molecular dynamics simulations (CMD) which purpose was to make the model systems to reach their thermodynamic equilibration states, and SMD, were carried out using the programs NAMD 2.6 [42] with constant molecular number, pressure, and temperature (NPT) and periodic boundary conditions. The CHARMM 27 force field [43], [44] was employed for the protein and the gaff force field [45] for imatinib. The model systems were solvated in a bath of TIP3P water molecules [46] with the buffering distance set to 15 Å which is large enough for the conformational changes. By assuming normal charge states of ionizable groups corresponding to pH 7, sodium (Na^+^) and chloride (Cl^−^) counter-ions at physiological concentration of 0.15 mol·L^−1^ were added in the box in random positions to ensure the global charge neutrality. All Na^+^ and Cl^−^ ions were placed more than 8 Å away from any protein atoms and from each other.

Constant temperature (T = 310 K) and constant pressure (P = 1 bar) were maintained using Langevin piston coupling algorithm [47]. The integration time step of the simulations was set to 2.0 fs, the SHAKE algorithm was used to constrain the lengths of all chemical bonds involving hydrogen atoms at their equilibrium values [48], and the water geometry was restrained rigid by using the SETTLE algorithm [49]. Non-bonded van der Waals interactions were treated by using a switching function at 10 Å and reaching zero at a distance of 12 Å. Long-range electrostatic forces were handled by using the particle-mesh Ewald (PME) algorithm [50], which is an efficient full electrostatics method for use with periodic boundary conditions.

The systems were firstly minimized using steepest-descents algorithm. Afterward, the systems were heated from 0 K to 310 K in two steps: the first step was a 100ps heating process with the protein and ligand fixed; the second step was another 100 ps heating process with the whole system relaxed. Thirdly, a 200 ps CMD simulation was performed with the protein and ligand fixed, followed by a 4 ns CMD simulation with the whole system relaxed. The SMD simulations were then performed, in which initial structures were the snapshots randomly taken from the equilibration states in the CMD simulations. The acting point of pulling point for the ATP-channel is the mass center of pyridine group of imatinib, and that for the allosteric-pocket-channel is the mass center of piperazinyl group of imatinib. The harmonic constraints were applied to the Cα atoms of helix in the Abl and c-Kit and the exponent to be used in the harmonic constraint energy functions was 2. The force constant and velocity used in the SMD simulations are 4 kcal mol^−1^ Å^−2^ (277.9 pN Å^−1^) and 0.00005 Å timestep^−1^ (0.025Å ps^−1^), respectively.

By the way, it is necessary to point out that the affinity of the ligand for the two pockets can not be directly given by the force measurements in the current SMD simulations, although SMD provides a possibility to calculate the binding affinity by, for example, applying the Jarzynski equality [36]. This is mainly due to that (1) the system concerned here experiences a large conformational change, and (2) the SMD simulations here are still very limited. To obtain the ligand binding affinity, a much larger set of simulations are required. Fortunately, the calculation of ligand binding affinity is not necessory for the objective in this investigation that is to explore the possible dissociation pathways of typical type II inhibitor imatinib from its targeting protein kinases c-Kit and Abl.

## Results and Discussion

### Dissociation of Imatinib from c-Kit

**Figure 3:**
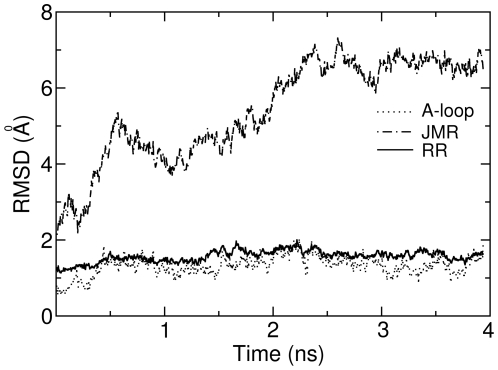
RMSD (root mean square deviation) profiles of key motifs in imatinib-c-Kit complex during conventional molecular dynamics simulation. The reference structure from which the RMSD was calculated is the crystal structure 1T46.

Before performing SMD simulations to pull imatinib out of c-Kit, 4 ns CMD simulation was first carried out which purpose was to make the whole system equilibrated at the designated temperature. Figure 3 shows the variations of RMSD values for the Cα atoms of JMR (TYR1-ASP33), A-loop (CYS205-LEU227) and the remaining regions (RR, that are all the residues of c-Kit except those in JMR and in A-loop), relative to the crystal structure during the MD simulations. Obviously, in the beginning, the RMSD values for the JMR have a significant change, which is understandable since JMR is a loop and completely exposed to the solvent. In contrast to JMR, a small fluctuation of RMSD values is observed for A-loop and RR. After 3 ns, the RMSD values for the whole system fluctuate steadily, implying that the whole system reaches its equilibration state.

After that, ten snapshots were randomly chosen from the equilibration state (3ns–4ns) as the initial structures for the subsequent SMD simulations, with five snapshots for each pathway (ATP-channel or allosteric-pocket-channel). Before executing the actual SMD simulations, all the possible directions for the pulling force were identified based on the relative orientation of ligand and its binding site. Then five independent SMD simulations for each pathway were performed, in which the direction of pulling force was randomly chosen in the range of possible directions just identified.. The simulation time for each SMD simulation is 1 ns.

**Figure 4:**
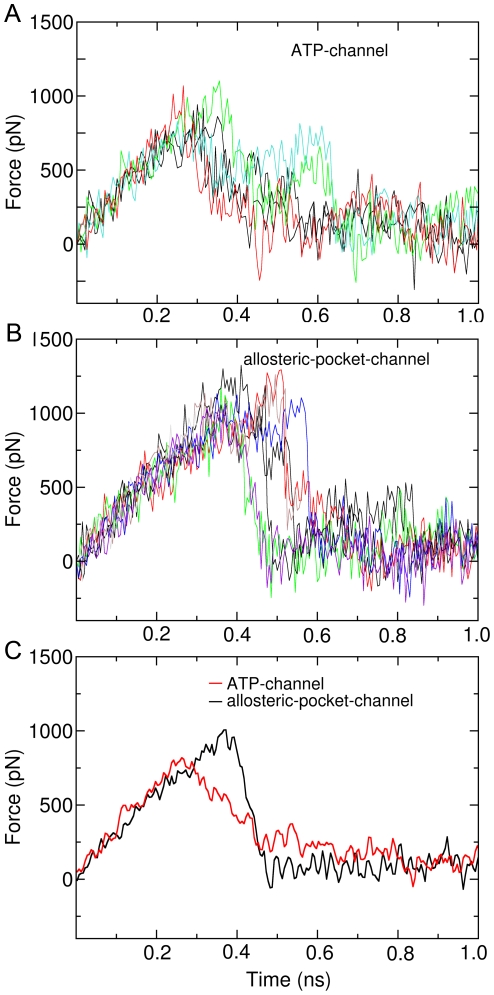
Force profiles of imatinib dissociation from imatinib-c-Kit complex. (A) Force profile of imatinib unbinding along traditional ATP-channel. (B) Force profile of imatinib unbinding along allosteric-pocket-channel. (C) Averaged force profiles of imatinib unbinding along ATP-channel (red) and allosteric-pocket-channel (black).

Figure 4A presents the profiles of pulling force during the SMD simulations for the ATP-channel. Clearly, increased force appears when the ligand imatinib begins to move out of the binding site, irrespective of initial structure and the direction of pulling force, which imply that the ligand encounters energy barriers. The maximum pulling forces for the ATP-channel are between 737–1080 pN, and averaged at 816 pN (see Figure 4C). Finally the pulling force becomes to zero, indicating that the ligand completely dissociated from c-Kit. The pulling force profiles for dissociation along the allosteric-pocket-channel are shown in Figure 4B. Very similar to the ATP-channel, the forces increase in the beginning of ligand unbinding, and reaches a maximum around 0.4 ns, followed by returning to zero.

Visibly, the ligand suffers a smaller force when dissociation along the ATP-channel than through the allosteric-pocket-channel. Quantitatively, the maximum average force along the ATP-channel is 816 pN, which is smaller by 19% than that along the allosteric-pocket-channel (1008 pN). The integrated forces shown in Table 1 reflect even clearer how dissociation is easier along ATP-channel: the total force required for the ligand to be extracted is 332 pN·ns, which is 16% smaller than that in allosteric-pocket-channel (397 pN·ns).

**Table 1:** Properties (maximum force and integrated force) of the average forces suffered by imatinib during dissociation processes along the ATP-channel and allosteric-pocket-channel.

		Maximum force (pN)	integrated force (pN·ns)
imatinib-c-Kit system	ATP-channel	816	332
	Allosteric-pocket-channel	1008	397
imatinib-Abl system	ATP-channel	780	324
	Allosteric-pocket-channel	830	384

What are the determining factors to result in that the ligand dissociation along the allosteric-pocket-channel is difficult than the ATP-channel? Here one may simply think that the JMR, which locates outside of the allosteric-pocket-channel, might be the most important factor. Thus, in order to further explore this matter, another model system, namely, imatinib-Abl, in which Abl is a cytoplastic kinase, was chosen to perform the same SMD simulation. Since no JMR in the Abl kinase, imatinib-Abl is expected to be a good model to judge the role of JMR.

### Dissociation of Imatinib from Abl

**Figure 5:**
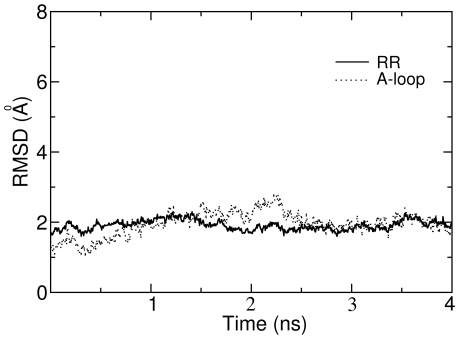
RMSD (root mean square deviation) profiles of key motifs in imatinib-Abl complex during conventional molecular dynamics simulation. The reference structure from which the RMSD was calculated is the crystal structure 2HYY.

Again a 4 ns CMD simulation was firstly performed in order to make the imatinib-Abl equilibrated in the solvent at the designated temperature. Figure 5 depicts the variation of the RMSD values relative to the initial structure for the Cα atoms of A-loop (ALA146-PRO168) and the remaining regions (RR, that are all the residues of Abl except those in A-loop). Clearly, after 2.5 ns, the RMSD values for A-loop, RR, and hence the overall system, fluctuate steadily, implying that the system reaches its equilibration state.

**Figure 6:**
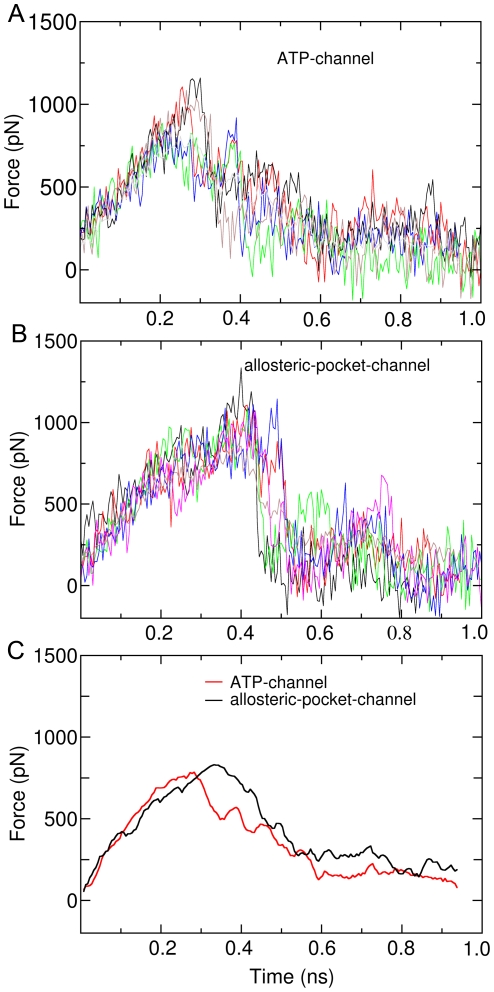
Force profiles of imatinib dissociation from imatinib-Abl complex. (A) Force profile of imatinib unbinding along ATP-channel. (B) Force profile of imatinib unbinding along allosteric-pocket-channel. (C) Averaged force profiles of imatinib unbinding along ATP-channel (red) and allosteric-pocket-channel (black).

Similarly, we selected randomly ten snapshots in the equilibration state as the initial structures for the SMD simulations. Five of them are for the ATP-channel and the other five for the allosteric-pocket-channel. Figure 6A and 6B show the pulling force profiles for dissociation along the ATP-channel and allosteric-pocket-channel, respectively. For both pathways, the pulling forces increase in the beginning of ligand unbinding process, irrespective of initial structure and the direction of pulling force, indicating again that the ligand encounters energy barriers. Finally the pulling forces return to zero, denoting that the ligand completely dissociated from the Abl.

Comparisons of the force profiles for both pathways show that the maximum pulling forces for the ATP-channel are obviously smaller than that for the allosteric-pocket-channel. The average forces together with the integrated forces shown in Figure 6C and Table 1 further clearly demonstrate that dissociation of imatinib is easier along ATP-channel than allosteric-pocket-channel: the total force required for the ligand to be extracted is 324 pN·ns, which is 16% smaller than that in allosteric-pocket-channel (384 pN·ns).

### Intrinsic Reasons Why the Ligand Dissociation through the ATP-Channel Is Favored

The abo**v**e simulations clearly indicate that the ligand imatinib dissociates from its binding protein kinases readily through the ATP-channel rather than the allosteric-pocket-channel, irrespective of the receptor, initial structure, and the direction of pulling force. Further, the simulation results also demonstrate that the JMR that resides outside of the allosteric-pocket-channel is not a factor that results in that the allosteric-pocket-channel is not preferred. What are the determining factors leading to that the ligand dissociation through the ATP-channel is favored? In order to address this question, we analyzed the key components of interaction energies between the ligand and protein kinases during SMD simulations, including electrostatic, van de Waals (vdW), and hydrogen-bonding interactions. It was found that the profiles of electrostatic and hydrogen-bonding interaction energy were very similar for the both pathways (data not shown). These are understandable due to the following reasons: (1) the ligand imatinib is a very weak polar molecule, which results in that the ligand suffers very similar electrostatic interaction when dissociating through different pathways; (2) hydrogen bonds are always in the courses of breaking and forming in the ligand unbinding process, and the overall number of hydrogen bonds keep relatively invariable.

**Figure 7:**
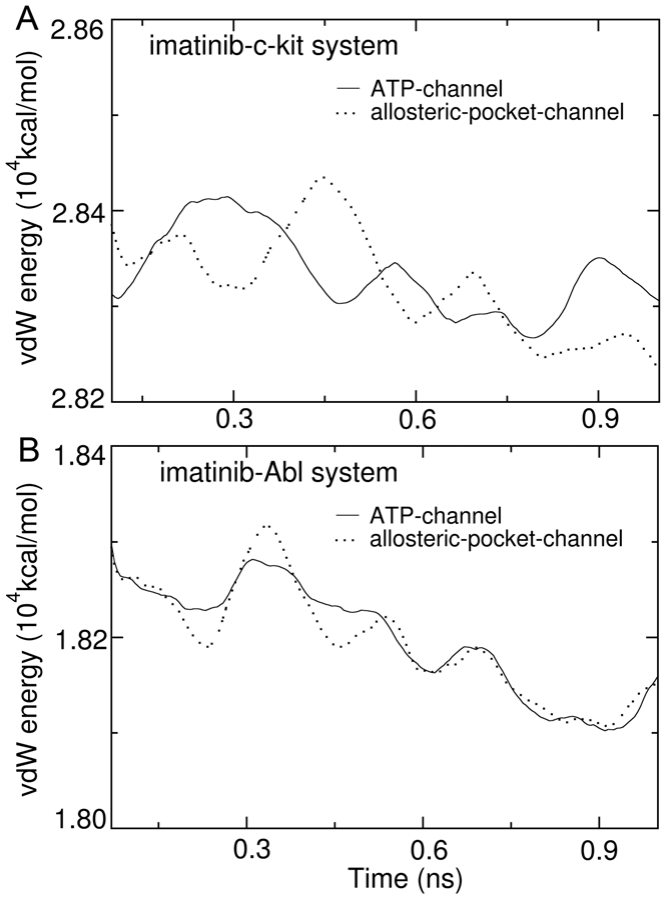
Energy profiles of van der Waals (vdW) interaction between imatinib and its targeting kinases during dissociation. (A) vdW interaction of imatinib-c-Kit system. (B) vdW interaction of imatinib-Abl system. The vdW profiles shown in solid line are for the ATP-channel and those shown in dotted line are for the allosteric-pocket-channel.

For the vdW interaction energy, however, obvious differences could be found for these profiles along the ATP-channel and allosteric-pocket-channel. Figure 7A presents the profiles of average vdW interaction energy between imatinib and c-Kit during dissociation through the two pathways. Obviously, there is some fluctuation for both of the vdW energy profiles during the SMD simulations. The highest vdW energy peak (the energy barrier), however, is on the curve corresponding to the allosteric-pocket-channel. The time point for the highest vdW energy peak is around 0.4 ns, which is consistent with the time when the ligand suffers the maximum pulling force (see Figure 4C). The vdW interaction energy profiles for the imatinib-Abl system during dissociation through ATP-channel and allosteric-pocket-channel are shown in Figure 7B. Again, a fluctuation is observed for both of the vdW energy curves during the SMD simulations. The highest vdW energy peak (the energy barrier) occurs on the profile corresponding to the allosteric-pocket-channel. The time point for the highest vdW energy peak is around 0.3 ns, which is also consistent with the time when the ligand suffers the maximum pulling force (see Figure 6C).

**Figure 8:**
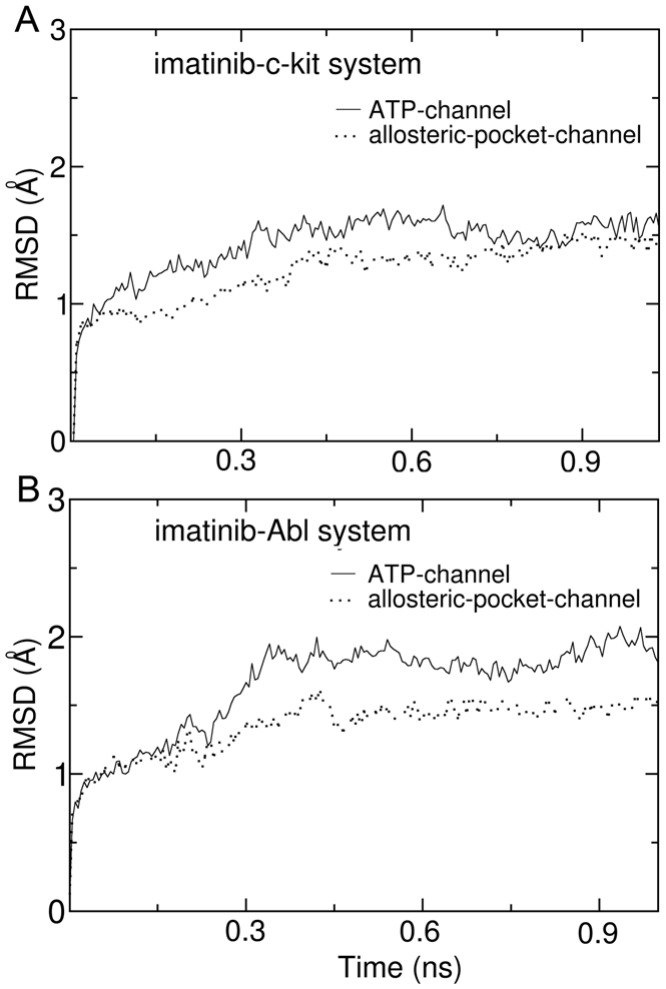
Variations of RMSD (root mean square deviation) values for the residues composing the ATP-channel (solid line) and allosteric-pocket-channel (dotted line) during the steered molecular dynamics simulations. (A) is for imatinib-c-Kit system, and (B) for imaintib-Abl system.

The preceding energy analysis indicates that the difference of vdW interaction energy is possibly a key factor determining that the ligand dissociation through the ATP-channel is favored. But what are the intrinsic reasons leading to the difference of vdW interaction energies? In order to answer this question, we further analyzed the flexibility of residues forming the ATP-channel and allosteric-pocket-channel. Figure 8A and 8B present the variations of RMSD values for the residues composing the ATP-channel and allosteric-pocket-channel during the SMD simulations. Very clearly, for both of the imatinib-c-Kit and imatinib-Abl model systems, the variations of RMSD values for ATP-channel residues are much larger than that for allosteric-pocket-channel, implying that the residues forming the ATP-channel are more flexible than that forming the allosteric-pocket-channel. These are consistent with B-factor values experimentally measured [see crystal structures of imatinib-c-Kit complex (PDB entry: 1T46) and imatinib-Abl complex (PDB entry: 2HYY)]: the values of B-factor for the residues forming the ATP-channel are generally larger than that for the residues forming the allosteric-pocket-channel. Further, the three-dimensional structures of ATP-channel and allosteric-pocket-channel provide additional evidences: the residues forming the ATP-channel belong to certain β-sheet or loop, nevertheless, in addition to β-sheets and loops, part of residues composing the allosteric-pocket-channel also belong to the α-helix (helix αC, see Figure 1). In general, flexibility of residues in α-helix is smaller than that in β-sheet and loop.

### Implications to the Development of Type II Kinase Inhibitors

**Figure 9:**
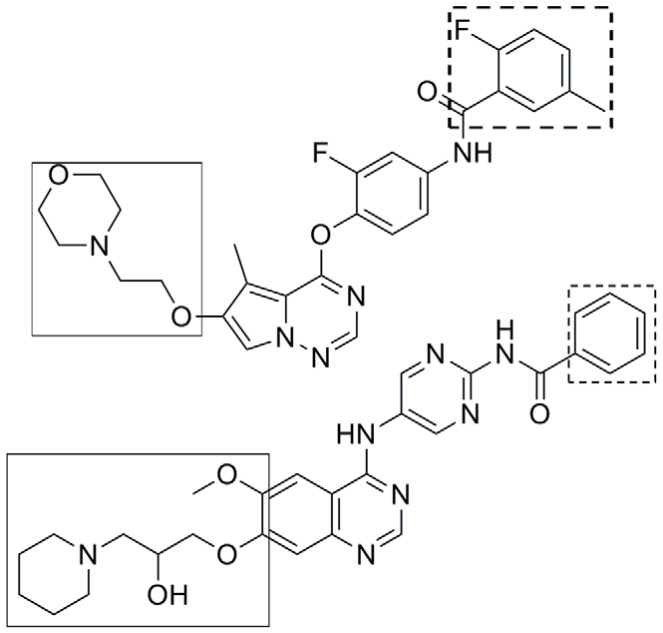
Chemical structures of selected type II kinase inhibitors. The substituent in dotted rectangle corresponds to the head of inhibitor and that in solid rectangle corresponds to the tail of inhibitor.

Finally, what implications to the development of type II protein kinase inhibitors could be obtained from the present investigation? As discussed above, the likelier dissociation pathway for the ligand from its targeting kinases corresponds to the ATP-channel. Rationally we can deduce that the ATP-channel would be also preferred for the ligand association. This implies that, in the association process of a type II kinase inhibitors, the hydrophobic head of the inhibitor that is the motif locating in the allosteric pocket, has to first pass through the ATP-channel and then the allosteric-pocket-channel. It is clear that a bulky hydrophobic head in type II kinase inhibitors is unfavorable. On the contrary, a bulky polar tail in type II kinase inhibitors is allowed. These agree very well with chemical structures of most of the known type II kinase inhibitors that they often have a smaller hydrophobic head. Several typical examples are shown in Figure 9.

In conclusion, molecular dynamics simulations reveal that the most favorable pathway for imatinib dissociation corresponds to the ATP-channel rather than the relatively wider allosteric-pocket-channel, which is mainly due to the different van der Waals interaction that the ligand suffers during dissociation. Nevertheless, the direct reason might come from the fact that the residues composing the ATP-channel are more flexible than those forming the allosteric-pocket-channel. This investigation, in addition to clarify the dissociation mechanism of type II kinase inhibitor imatinib from its targeting protein kinases, also suggests us that a bulky hydrophobic head is unfavorable, but a large polar tail is allowed for a potent type II inhibitor. The information obtained here can be used to direct the discovery of type II kinase inhibitors.
